# Supplementary material for: Enhancing Functionalization of Health Care Textiles with Gold Nanoparticle-Loaded Hydroxyapatite Composites
Source: Nanomaterials (Basel). 2023 May 27;13(11):1752. doi: 10.3390/nano13111752 (PMC10254177; doi:10.3390/nano13111752)
Supplement: Supplementary file 1 [file nanomaterials-13-01752-s001.zip › nanomaterials-2378620-supplementary.pdf]

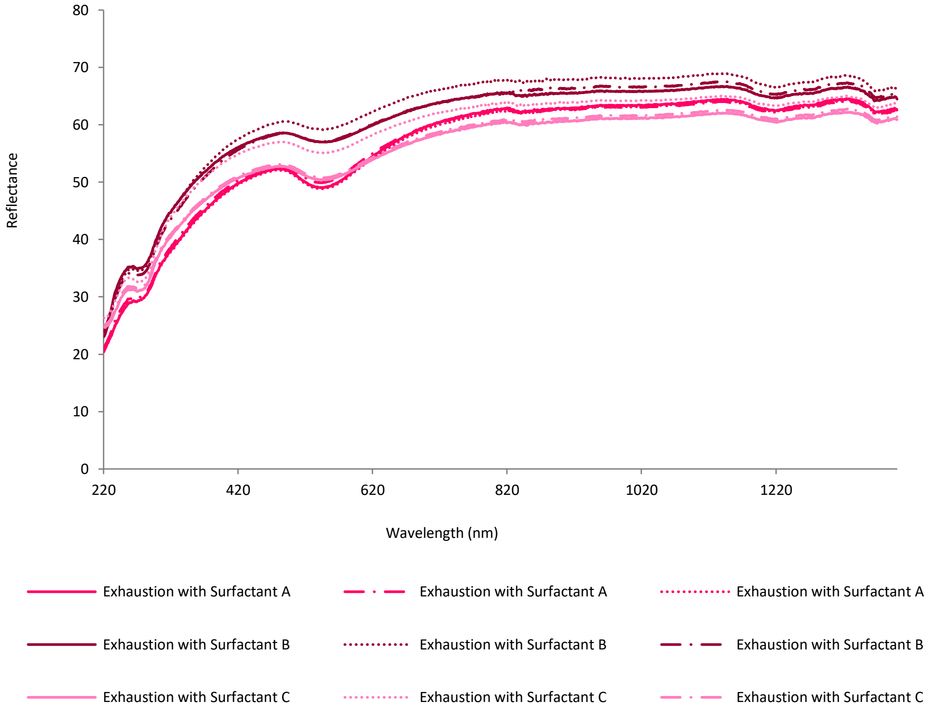

Figure S1. Reproducibility of the replications performed for each textile functionalized sample (with 0.1 mg/mL of AuNPs dispersion and 4 g/L of surfactant A or 0.5 g/L of surfactant B or 3 g/L of surfactant C, by exhaustion at 60 °C for 60 minutes) during the reflectance readings in the UV-Vis equipment.

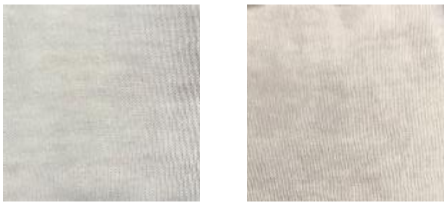

Figure S2. Cotton substrates functionalized with 0.1 mg/mL AuNPs. **Left**: Exhaustion with surfactant A + surfactant D; **Right**: Exhaustion with surfactant B + surfactant D.

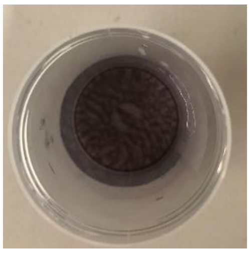

Figure S3. Master dispersion with AuNPs and Surfactant B + surfactant D.

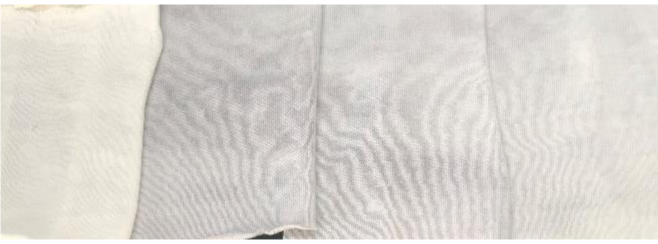

Figure S4. Samples functionalized with different RB (exhaustion with 0.1 mg/mL of AuNPs and 4 g/L of surfactant A, at 60 °C for 60 min). From left to right: Control sample without NPs; exhaustion with 20:1 ratio; exhaustion with 12:1 ratio and exhaustion with 7:1 ratio.

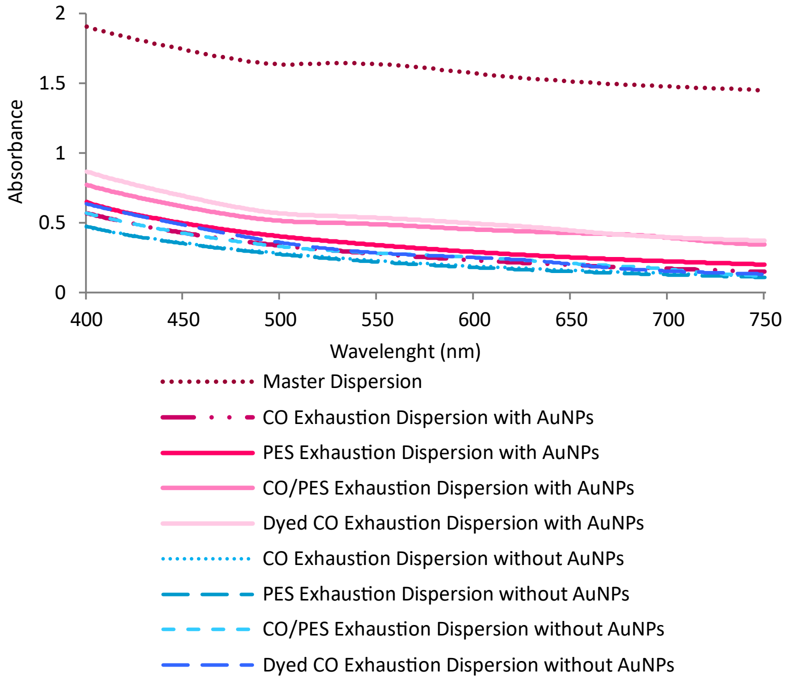

Figure S5. Absorbance of dispersions after exhaustion processes (with 0.1 mg/mL of AuNPs and 4 g/L of surfactant A and 5 g/L of surfactant D, at 70 °C for 10 min) in different substrates.
